# *Mycobacterium tuberculosis* Heat-Shock Protein 16.3 Induces Macrophage M2 Polarization Through CCRL2/CX3CR1

**DOI:** 10.1007/s10753-019-01132-9

**Published:** 2019-11-20

**Authors:** Yanhao Zhang, Shanshan Li, Qianyi Liu, Ruiying Long, Jihong Feng, Huan Qin, Mao Li, Liping Liu, Junmin Luo

**Affiliations:** Department of Immunology, Zunyi Medical University, No. 6, Xuefu West Road, Xinpu District, Zunyi, 563000 Guizhou China

**Keywords:** *Mycobacterium tuberculosis*, macrophage polarization, *Mycobacterium tuberculosis* heat-shock protein 16.3, chemokine receptors

## Abstract

*Mycobacterium tuberculosis*, the pathogen of tuberculosis (TB), can survive in host macrophages and induce macrophages to M2 phenotype might result in latent MTB infection. During the latent phase, the expression of MTB heat-shock protein 16.3 (Hsp16.3) is markedly increased among most of bacterial proteins, but the role of Hsp16.3 in macrophage M2 polarization is not clear. In this work, we found that macrophages incubated with 100 ng/ml MTB Hsp16.3 increased the production of Arg-1, IL-10, TGF-beta, and CD206. These results showed that MTB Hsp16.3 may induce macrophage M2 phenotype. And the interaction of Hsp16.3 with macrophages was found to depend on chemokine receptors CCRL2 and CX3CR1. Additionally, we used overexpression and silencing techniques to further verify the effect of CCRL2 and CX3CR1 on MTB Hsp16.3-induced M2 polarization macrophages. Furthermore, we explored the downstream signaling molecules of CCRL2 and CX3CR1 and we found MTB Hsp16.3 altered the signal transduction of the AKT/ERK/p38-MAPK. Taken together, this study provides evidence that MTB Hsp16.3 promotes macrophages to M2 phenotype and explores its underlying mechanism.

## INTRODUCTION

Tuberculosis (TB) poses a serious threat to human health. According to the WHO, the number of new TB cases in 2017 was approximately 10 million, and the global death rate is approximately 1.57 million [1]. *Mycobacterium tuberculosis* (MTB), an intracellular pathogen, causes TB and leads to both latent and acute infections. MTB usually enters the body through inhalation into the lungs, and alveolar macrophages phagocytose the bacteria and move to the pulmonary interstitial space where they recruit mononuclear-derived macrophages and other immune cells to form tuberculous granuloma. It is generally considered that the main function of the tuberculous granuloma is to localize the infecting bacteria and prevent the spread of infection [2], but MTB uses this opportunity to inhabit macrophages and survive for a prolonged time through their own components and virulence factors [3].

Macrophages that differentiate from monocytes and bone marrow hematopoietic stem cells are the first line of defense against microbes; they have cytokine secretion, phagocytosis, and antigen presentation functions. Macrophages exhibit vast functional plasticity and polarize to a certain phenotype in response to the microenvironment. Due to their functional characteristics and morphology, macrophages can be classified into classically activated macrophages (or M1) and alternatively activated macrophages (or M2). M1 macrophages, polarized by interferon-gamma (IFN-γ) and lipopolysaccharide (LPS), have increased expression levels of inflammatory cytokines (interleukin (IL)-12, tumor necrosis factor-α (TNF-α), and IL-6) [4], inducible nitric oxide synthase (iNOS), and CD86, and have the capacity to kill pathogens and tumor cells, and promote a Th1-type immune response.

In contrast, M2 macrophages are associated with anti-inflammatory activity. According to different stimuli, M2 phenotype macrophages can be further divided into three subpopulations, M2a, M2b, and M2c. M2a macrophages induced by IL-4 and IL-13 express the mannose receptor, scavenger receptor, and arginase-1 (Arg-1); M2b macrophages, stimulated by IL-1 receptor ligands and immune complexes, increase the production of IL-10 and promote the Th2 response, whereas M2c macrophages, mainly induced by IL-10, secrete pentraxin 3 and chitinase 3-like 3 and play a role in wound healing [5].

Research has shown that macrophages play a dual role in MTB infection. On the one hand, macrophages are the main immune effector cells and antigen-presenting cells responsible for the anti-tuberculosis response; on the other hand, macrophages are a habitat of *M. tuberculosis*, and they provide protection to MTB [6]. MTB has evolved several strategies to reside and even replicate within macrophages, including preventing phagosome-lysosome fusion, inhibiting phagolysosomal maturation, and inducing M2 macrophage polarization [2]. A study showed that M2 phenotype macrophages contribute to the creation of a suppressive microenvironment that can promote MTB intracellular growth and latent infection. Therefore, an improved understanding of macrophage polarization during MTB infection may be helpful to cure TB.

Recent studies have found that small heat-shock proteins (sHsps) play an important role in the development of tuberculosis. sHsps, which maintain cell stability, aid denatured protein refolding, and participate in cell damage and repair, are present in most organisms. MTB heat-shock protein 16.3 (Hsp16.3), a member of the α-crystal superfamily, is encoded by the HspX gene and is known as Acr1, 16-kDa antigen, and Rv2031c. Hsp16.3 has a relative molecular weight of approximately 16.3 kDa. MTB invades the body and is phagocytosed by pulmonary macrophages; in order for MTB to survive in the macrophage environment, DosR gene expression is activated, which upregulates the expression of many bacterial proteins, of which MTB Hsp16.3 is the highest [7, 8]. Studies have shown that MTB Hsp16.3 impacts macrophage apoptosis and autophagy [9]. All of the above studies suggest that MTB Hsp16.3 plays a role in the survival of host macrophages during latent infection.

In our previous work, we constructed a prokaryotic expression vector, pET28a-MTB Hsp16.3, which induces the expression of MTB Hsp16.3 and purified the fusion protein through nickel affinity chromatography [10]. We found that MTB Hsp16.3 can induce M2 phenotype polarization in RAW264.7 cells. Incubation of macrophages with Hsp16.3 recombinant protein increases the secretion of TGF-β and IL-10, and compared with untreated macrophages; treated macrophages became long and stringy with increased numbers of pseudopods. MTB Hsp16.3 can induce the polarization of macrophages to the M2 phenotype, but the mechanism by which Hsp16.3 regulates macrophage polarization remains to be elucidated. In this study, we found that Hsp16.3 can induce the bone marrow-derived macrophage (BMDM) M2-like phenotype *via* CCRL2/CX3CR1 and may be mediated by the AKT/ERK/p38-MAPK signaling pathway.

## MATERIALS AND METHODS

### Animals

Female BALB/c mice (6–8 weeks old) were from the Experimental Animals Center of Chongqing Medical University, China. All the animals were housed in specific pathogen-free environment with regular food and water.

### Plasmid Construction

pcDNA3.1-CCRL2 (termed p-CCRL2) and pcDNA3.1-CX3CR1 (termed p-CX3CR1) plasmids were constructed. The CCRL2 and CX3CR1 genes were amplified by RT-PCR from the mRNA of macrophages derived from BALB/c mice (CCRL2: 5′-CGGGATCCATGGATAACTACACAGTGGCC C-3′ (forward), 5′-CCCAAGCTTTTATATTATATCCTGCCTTTGATGCA-3′ (reverse). CX-3CR1: 5′-CGGGATCCATGTCCACCTCCTTCCCTG-3′ (forward), 5′-CCCAAGCTTTCAGAGCAGGAGAGA CCCAT-3′ (reverse). The eukaryotic expression vector pcDNA3.1-CCRL2 and pcDNA3.1-CX3CR1 products were digested with BamHI and HindIII, respectively. Positive clone identity was verified by plasmid DNA sequencing (Invitrogen, Shanghai, China). Endotoxin-free plasmids were obtained using an Endofree Plasmid Mega kit (QIAGEN GmbH, Hilden, Germany). Then, plasmids were transiently transfected into the macrophages using Lipofectamine 2000 (Invitrogen, Shanghai, China) according to the manufacturer’s instructions.

### Cell Culture and Transfection

Bone marrow (BM) cells were isolated from the tibias and femurs of BALB/c mice, and the cells were cultured in Dulbecco’s modified Eagle’s medium (DMEM) low glucose (Gibco) supplemented with 10% FBS and 20 ng/ml GM-CSF. Cells were harvested on day 7 for further experiments. BM cells were identified by flow cytometry with an anti-mouse CD11b (APC) antibody (cat. no. 17-0112-82, eBioscience) and an anti-mouse F4/80 (FITC) antibody (cat. no. 11-4801-82, eBioscience). Cells were cultured in a humidified atmosphere of 5% CO_2_ at 37 °C. In terms of cell transfection, BMDMs were inoculated at a density of 60~80% before the transfection experiment. After 24 h, cells were transiently transfected with the indicated vectors with Lipofectamine 2000 according to the manufacturer’s instructions. Then, the cells were harvested at different time points.

### RNAi Experiments

Monolayers of cells cultured to approximately 70% confluency were subjected to siRNA transfection using the protocol recommended by the manufacturer (Ribort). Real-time PCR analysis was used to quantify the expression levels of siRNA-targeted genes.

### Real-Time PCR Assay

Total RNA was extracted from the cells using TRIzol (TAKARA, Beijing, China) according to the manufacturer’s instructions, and then total RNA was reverse transcribed to cDNA using a PrimeScript RT Master Mix kit (TAKARA, Beijing, China) following the manufacturer’s instructions. The primers and protocol used for amplification were as follows:

GAPDH, 5′-GAGCCAAACGGGTCATCATCT-3′ (forward), 5′-GAGGGGCCATCCACAGTC TT-3′ (reverse); TNF-α, 5′-CAGGGGCCACCACGCTCTTC-3′ (forward), 5′-TTTGTGAGTGTGAGGGTCT GG-3′ (reverse); IL-10, 5′-TACAGCCGGGAAGACAATAA-3′ (forward), 5′-AGGAGTCGGTTAGCA GTATG-3′ (reverse); TGF-β, 5′-GGCGGTGCTCGCTTTGTA-3′ (forward), 5′-TCCCGAATGTCTGA CGTATTGA-3′ (reverse); iNOS, 5′-CTGCAGCACTTGGATCAGGAACCTG-3′ (forward), 5′-GGAGT AGCCTGTGTGTGCACCTGGAA-3′ (reverse); Arg-1, 5′-CAGAAGAATGGAAGAGTCAG-3′ (forward), 5′-CAGATATGCAGGGAGTCACC-3′ (reverse); YM-1, 5′-GCAGAAGCTCTCCAATCCTG-3′ (forward), 5′-ATTGGCCTGTCCTTAGCCCAACTG-3′ (reverse); Fizz1, 5′-GCTGATGGTCCCAGTG AAAC-3′ (forward), and 5′-CCAGTAGCAGTCATCCCAGC-3′ (reverse). Real-time PCRs were performed in a BIO-RAD CFX96 detection system (Bio-Rad Laboratories) using a SYBR Premix Ex TaqTM kit (TAKARA, Beijing, China). The standard PCR conditions consisted of 95 °C for 30 s, followed by 40 cycles of 95 °C for 5 s and 60 °C for 34 s, with a final dissociation stage. The relative expression of the indicated genes was calculated using the comparative threshold cycle (Ct) method.

### Flow Cytometry

BMDMs were stained with an anti-mouse F4/80 (FITC) antibody (cat. no. 11-4801-82, eBioscience), an anti-mouse CD206 (APC) antibody (cat. no. 17-2061-82, eBioscience), and an anti-mouse NOS2 (PE) antibody (cat. no. 25-5920-82, eBioscience) for 30 min at 4 °C and then washed with PBS and analyzed in a flow cytometer (Beckman Coulter, USA).

### ELISA

Cell culture supernatant was collected, and then TNF-α, IL-10, and TGF-β levels were determined by an ELISA kit (eBioscience, CA) according to the manufacturer’s instructions. Standard curves of these cytokines were obtained using the recombinant standard proteins provided by the manufacturer.

### Western Blot Analyses

Western blotting was performed on cytosolic cellular extracts. Cells were washed with cold phosphate-buffered saline and lysed for 15 min on ice in 0.5 ml of lysis buffer containing protease and phosphatase inhibitors. Cell lysates were clarified by centrifugation (4 °C, 15 min, 12,000 rpm), and protein was subjected to 10% sodium dodecyl sulfate-PAGE (SDS-PAGE) and transferred to a nitrocellulose membrane using a wet transfer system. Membranes were incubated with 5% skim milk dissolved in TBS plus 0.05% Tween 20 (TBST) for 1 h to block nonspecific protein-binding sites. Then, the membranes were incubated with anti-CCRL2 (Abcam, no. ab88632), anti-CX3CR1 (Abcam, no. ab8021), anti-p38 MAPK (Abcam, no. ab197348), anti-ERK (Abcam, no. 196883), anti-AKT (Abcam, no. ab8805), anti-phospho-ERK (Abcam, no. ab50011), anti-phospho-AKT (Abcam, no. ab81283), anti-phospho-p38-MAPK (Abcam, no. ab47363), and anti-GAPDH (Abcam, no. ab181602) antibodies at 4 °C overnight according to the manufacturer’s instructions. The membranes were then washed with TBST and incubated with a secondary anti-rabbit Ab conjugated to HRP (Cell Signaling Technology, no. 7074s) at room temperature. The signals were detected and analyzed by a chemiluminescence imaging system (ChemiScope5600, CLINX, Shanghai, China), and each experiment was performed in triplicate.

### Statistical Analysis

The data are presented as the mean ± SD. An unpaired Student’s *t* test was used to evaluate differences between two groups, and a Student’s *t* test was then performed when two conditions were compared, and one-way ANOVA was performed for multiple comparisons followed by a post hoc Tukey’s test when necessary. If not, data were analyzed by the Mann-Whitney *U* test. *p* values of < 0.05 were considered significant; two-sided tests were performed. All data analyses were carried out by SPSS 16.0 (SPSS Inc.) and GraphPad Prism™ 5.0 (GraphPad Software Inc.) Statistical software.

## RESULTS

### MTB Hsp16.3 Induces Mouse Bone Marrow-Derived Macrophage M2 polarization

**Fig. 1:**
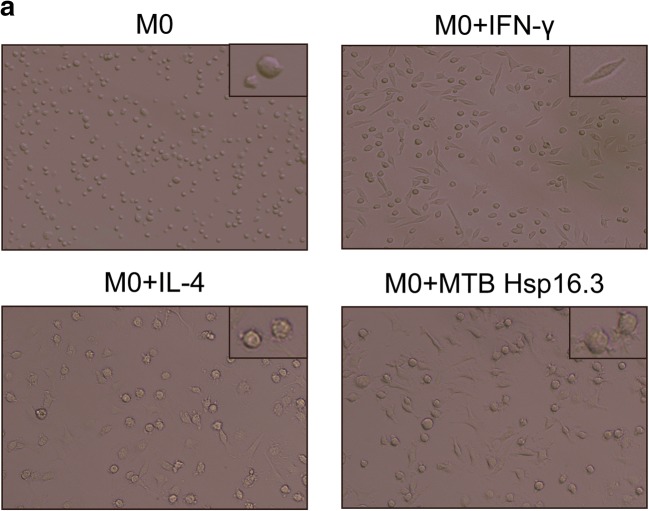

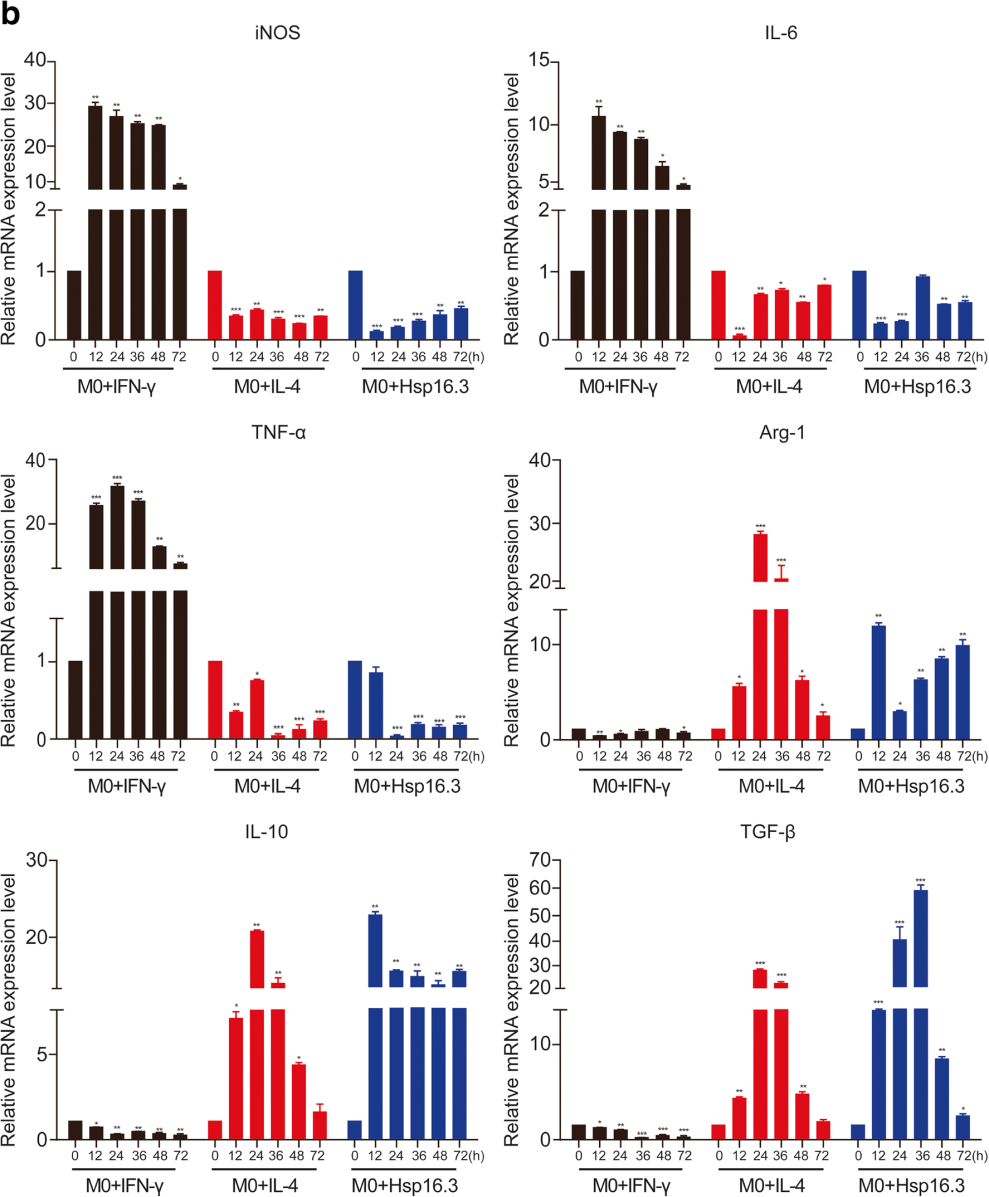

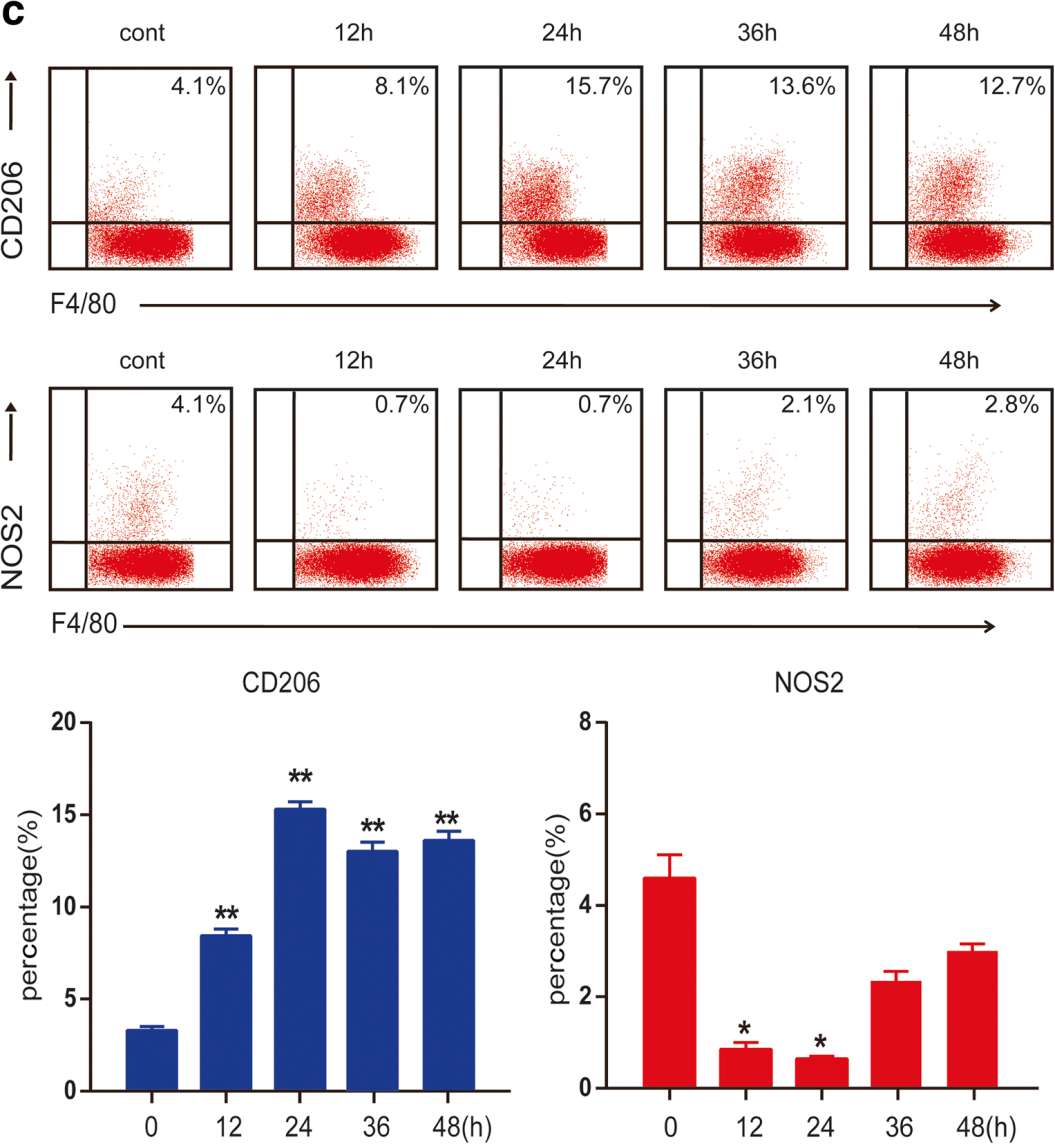

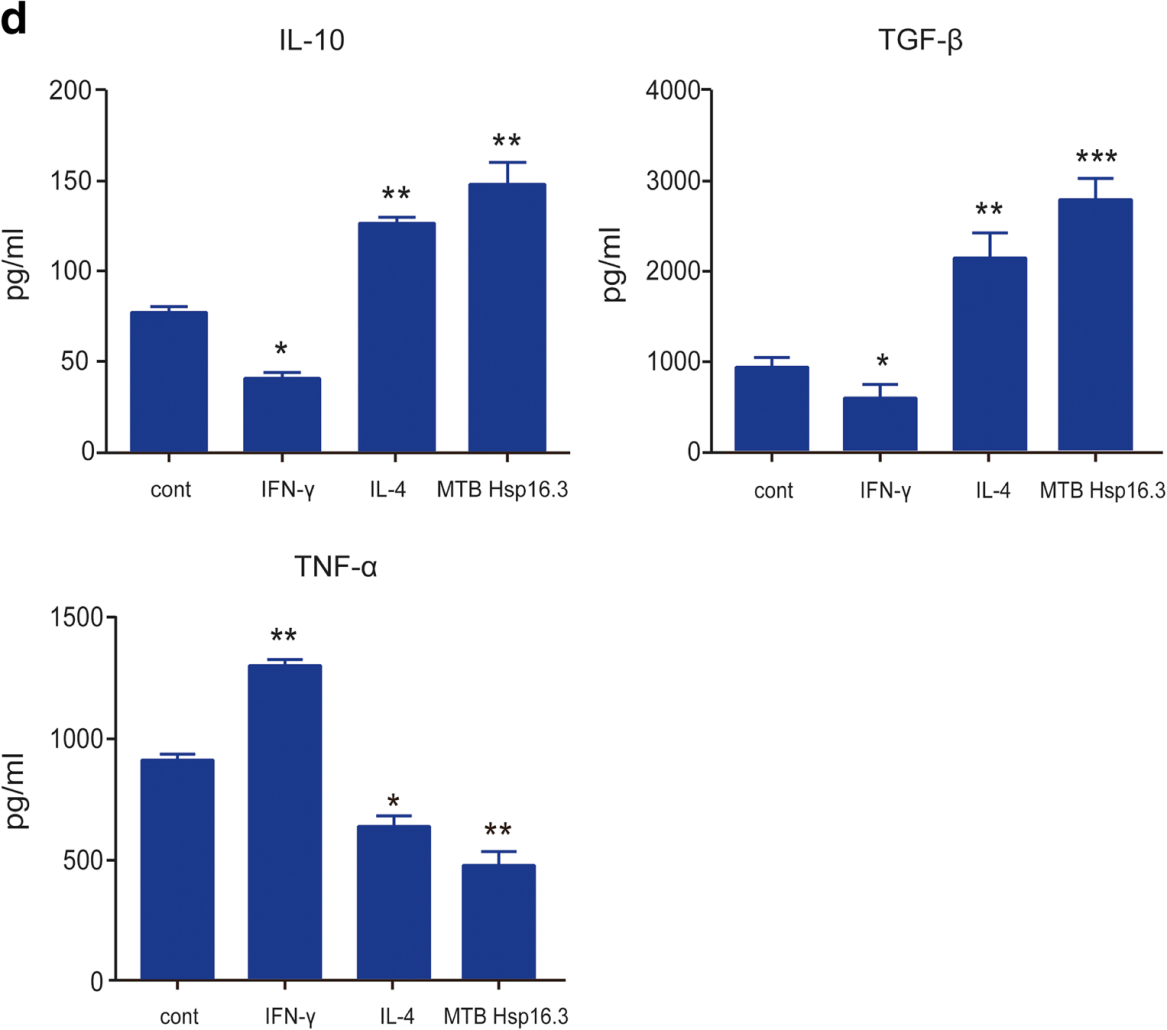
MTB Hsp16.3 induces mouse bone marrow-derived macrophage (BMDM) M2 polarization. Bone marrow cells were isolated from the tibias and femurs of BALB/c mice (6–8 weeks old) and incubated with 20 ng/ml GM-CSF for 7 days. Then, BMDMs were treated with 100 ng/ml MTB Hsp16.3, 100 ng/ml IFN-γ, or 100 ng/ml IL-4 for 0–72 h. **a** The morphology of BMDMs incubated with IFN-γ, IL-4, or MTB Hsp16.3 for 12 h. The images were captured under an inverted microscope (× 200); the picture in the upper right corner is × 400. Total RNA was extracted from the cells using TRIzol according to the manufacturer’s instructions. **b** The mRNA expression levels of iNOS, IL-6, TNF-α, Arg-1, IL-10, and TGF-β in macrophages by RT-PCR. **c** The percentage of F4/80 and NOS2 or CD206 double-positive macrophages was measured by FCM. **d** The production of TNF-α, IL-10, and TGF-β was measured by ELISA. Data are expressed as the mean ± SEM (*n* = 3). *p < 0.05, **p < 0.01, ***p < 0.001 compared with the control group (0 h group).

Bone marrow cells were cultured with GM-CSF for 7 days, and CD11b and F4/80 were detected by flow cytometry, showing 90.7% of cells to be double positive. The results showed that BMDMs were induced successfully (not shown in the figure). To verify the impact of MTB Hsp16.3 on macrophage polarization, BMDMs were incubated with 100 ng/ml IFN-γ, 10 ng/ml IL-4, and 100 ng/ml MTB Hsp16.3 for 12 h, 24 h, 36 h, 48 h, and 72 h. First, we observed the morphology of macrophages. Compared with the untreated macrophages (Fig. 1a), M1 macrophages (incubated with IFN-γ) had an elongated fibroblast-like morphology, whereas BMDMs incubated with MTB Hsp16.3 extended pseudopodia to the same extent as M2 macrophages (incubated with IL-4). Therefore, we speculated that MTB Hsp16.3 may induce the M2 phenotype in macrophages. Studies have reported that macrophages polarized to different phenotypes exhibit a distinct gene expression signature that can be used to identify them [11]. M1 phenotype macrophages secrete the inflammatory cytokines TNF-α, IL-12, IL-6, and iNOS. M2 phenotype macrophages produce IL-10 and TGF-β and show increased Arg-1 expression. In addition, M2 macrophage polarization can be defined based on a specific genetic signature characterized by the upregulation of Ym1 and Fizz1 expression. To verify which phenotype MTB Hsp16.3 treatment induces, we determined the expression of macrophage phenotype related markers. The mRNA expression levels of Arg-1, TGF-β, IL-10, iNOS, TNF-α, and IL-6 were determined by real-time PCR. The results showed that the Hsp16.3-treated group had significantly reduced expression levels of iNOS, TNF-α, and IL-6 mRNA (Fig. 1b) and upregulated expression levels of Arg-1, TGF-β, and IL-10 mRNA (Fig. 1b), as did the IL-4-treated BMDMs (M2 macrophages). We also found that the expression levels of Arg-1 and IL-10 peaked at 12 h, and TNF-α expression peaked at 24 h. Compared with those in the untreated group, the expression levels of iNOS and IL-6 in the treated group were suppressed at 12 h, and TGF-β expression was suppressed at 36 h. To investigate the macrophage surface molecules, flow cytometry was used to measure the expression levels of CD206 and NOS2. The +MTB Hsp16.3 group exhibited a markedly attenuated percentage of F4/80 and NOS2 double-positive cells (Fig. 1c). The percentage of F4/80 and CD206 macrophages was significantly increased in the MTB Hsp16.3 group compared with the untreated group (Fig. 1c). Then, we assessed if the M2-like cytokines had the same changes. ELISAs were performed to determine the expression of TNF-α, IL-10, and TGF-β (Fig. 1d). The results were as we expected, compared with untreated group, because the +MTB Hsp16.3 group showed increased production of IL-10 and TGF-β and reduced secretion of TNF-α. These results indicate that macrophages incubated with MTB Hsp16.3 recombinant protein show markedly upregulated expression of M2 phenotype markers.

### Genome-wide Microarray Analysis of BMDMs Incubated with MTB Hsp16.3

**Fig. 2:**
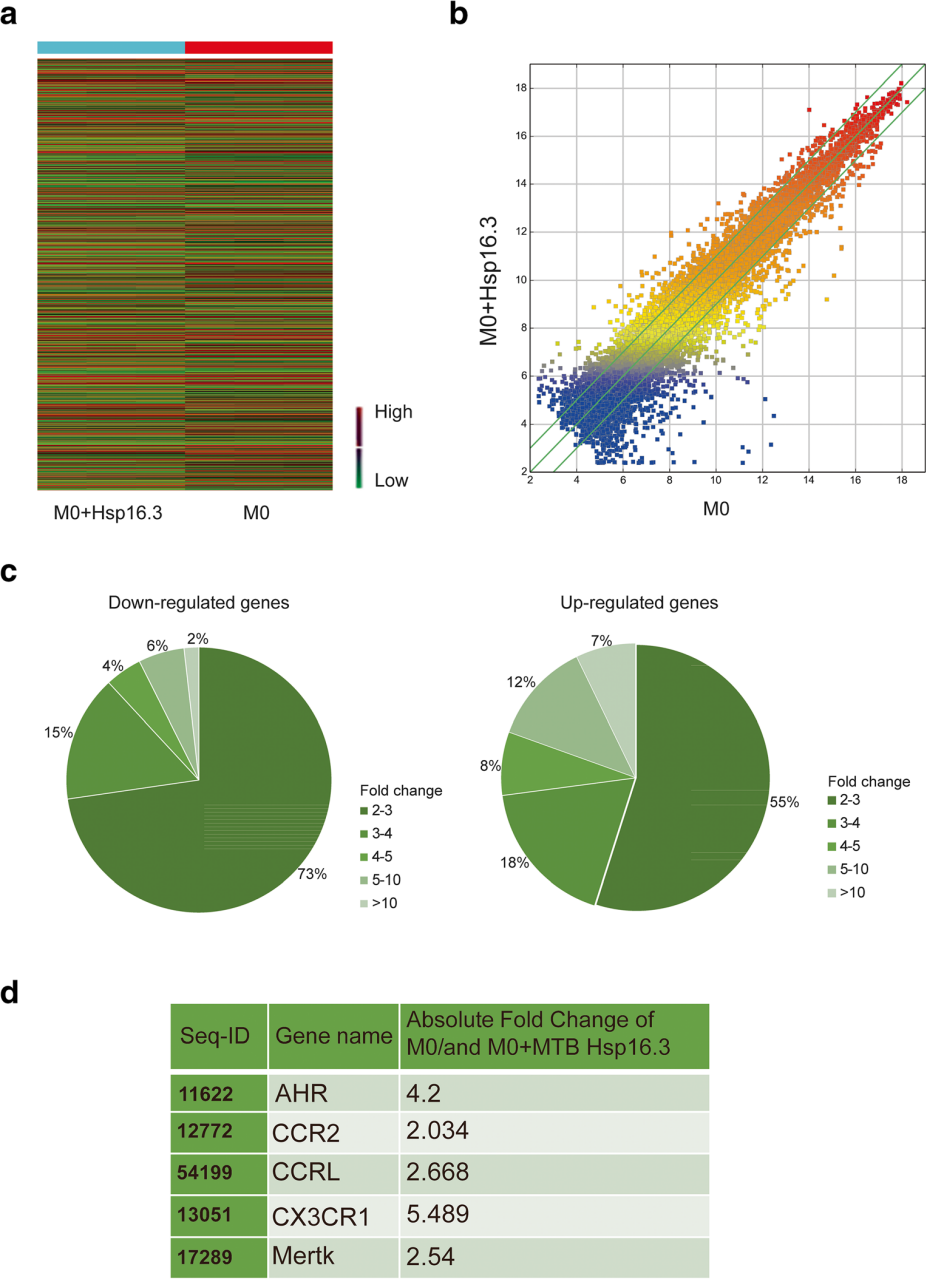

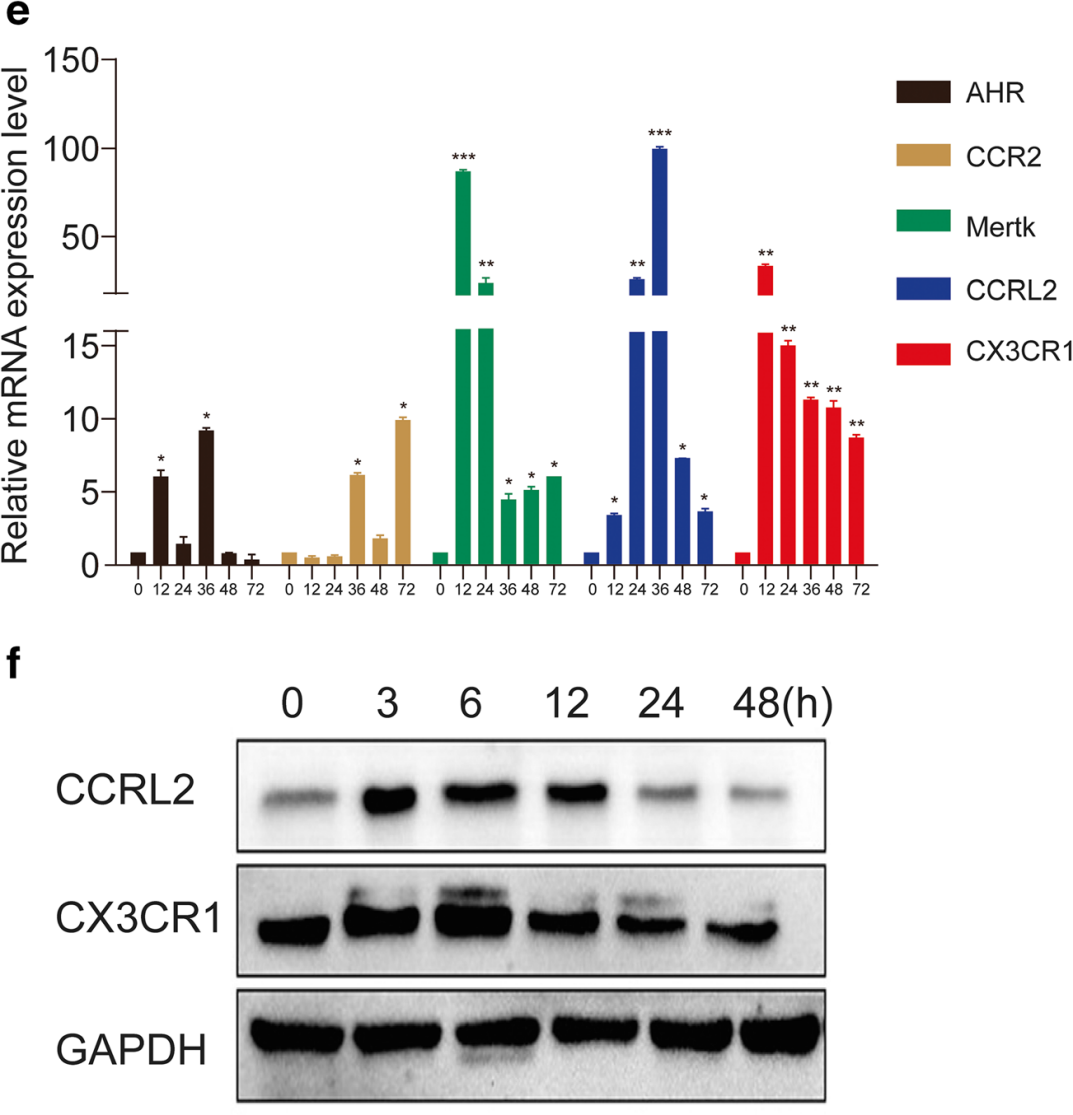
Genome-wide microarray analysis of BMDMs incubated with MTB Hsp16.3. Bone marrow cells were isolated from the tibias and femurs of BALB/c mice (6–8 weeks old) and incubated with 20 ng/ml GM-CSF for 7 days. Then, BMDMs were treated with 100 ng/ml MTB Hsp16.3 for 72 h, and the cells were collected. Global gene expression was analyzed by a cDNA chip array. **a** Heat map. **b** Scatterplot of gene expression. **c** The fold change and frequency. **d** Prediction of 5 target genes, including AHR, CCR2, CCRL2, CX3CR1, and Mertk. **e** BMDMs were treated with 100 ng/ml MTB Hsp16.3 for 72 h, cells were collected, and the mRNA expression of the indicated genes was determined by real-time PCR. **f** Cells were harvested in RIPA buffer containing protease and phosphatase inhibitor cocktails, and the protein expression levels of CCRL2 and CX3CR1 were determined by Western blot. Data are presented as the mean ± SEM (*n* = 3). *p < 0.05, **p < 0.01, ***p < 0.001 compared with the control group (0 h group).

To explore which receptor is affected by Hsp16.3 to promote BMDM M2 polarization, a genome-wide microarray was performed to investigate the variation in gene expression between the untreated group and the +Hsp16.3 group. The results showed that the gene expression of the two groups was relatively discrete (Fig. 2a, b). The number of genes with twofold upregulated expression was 568, and the number with twofold downregulated expression was 534 (Fig. 2c). To further elucidate the potential molecular mechanism by which Hsp16.3 induces macrophage M2 phenotype polarization, we examined the related literature and identified 5 genes, including CCRL2, CCR2, CX3CR1, AhR, and Mertk [12–16]. The results revealed that the expression of these genes changed approximately twofold between the two groups (Fig. 2d). We verified the expression of these 5 predicted target genes. We found that the CX3CR1 and CCRL2 mRNA expression levels were significantly upregulated in the +Hsp16.3 and more than 5 times greater than those the untreated group from 12 to 48 h (Fig. 2e). We also investigated the production of CX3CR1 and CCRL2, which peaked from 3 to 6 h in the treated group compared with the untreated group (Fig 2f). Collectively, our data indicate that Hsp16.3 might upregulate the expression of CX3CR1 and CCRL2 to polarize the macrophage M2 phenotype.

### Silencing of CCRL2/CX3CR1 Abrogates the MTB Hsp16.3-Induced Polarization of Macrophages to the M2 Phenotype

**Fig. 3:**
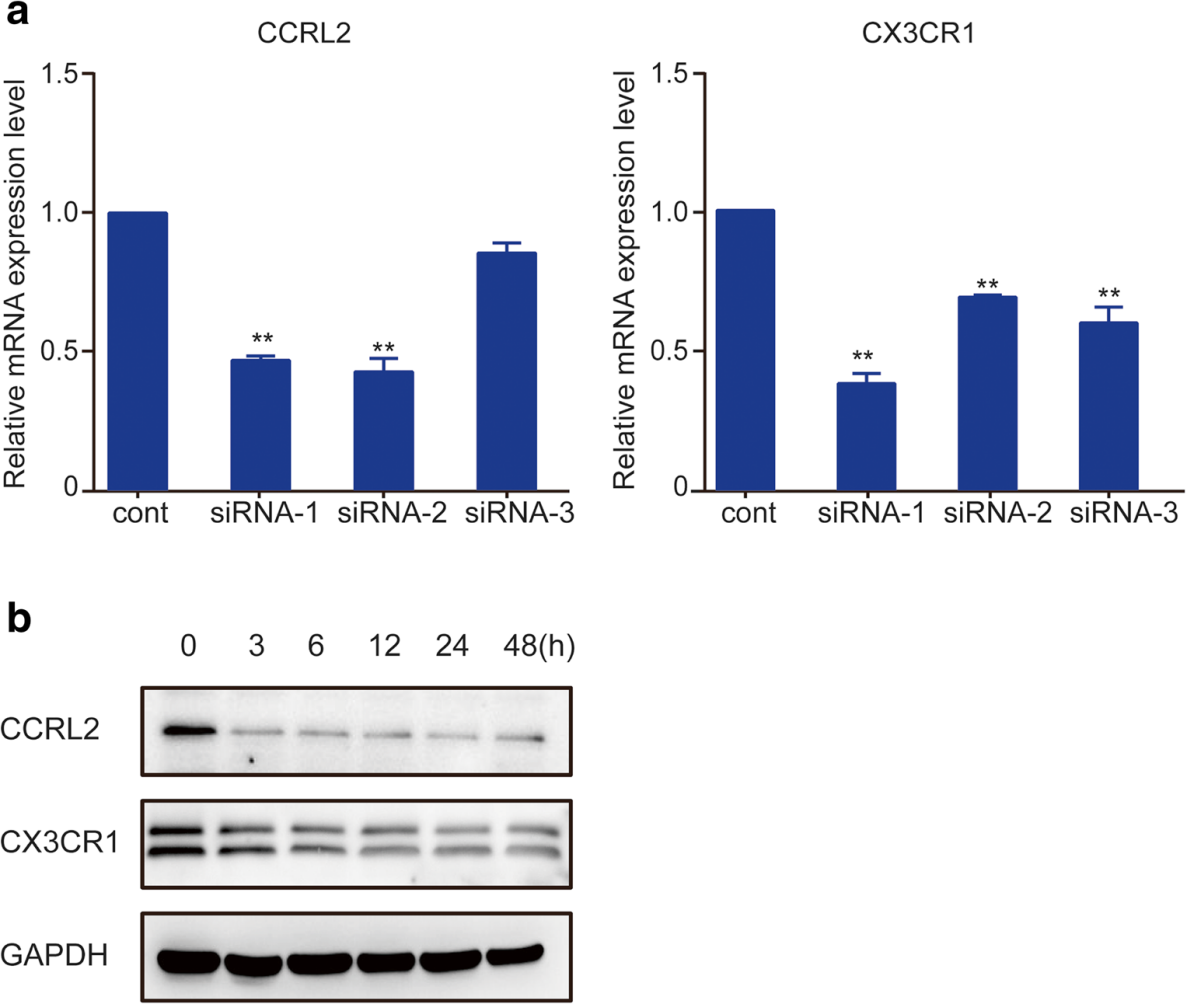

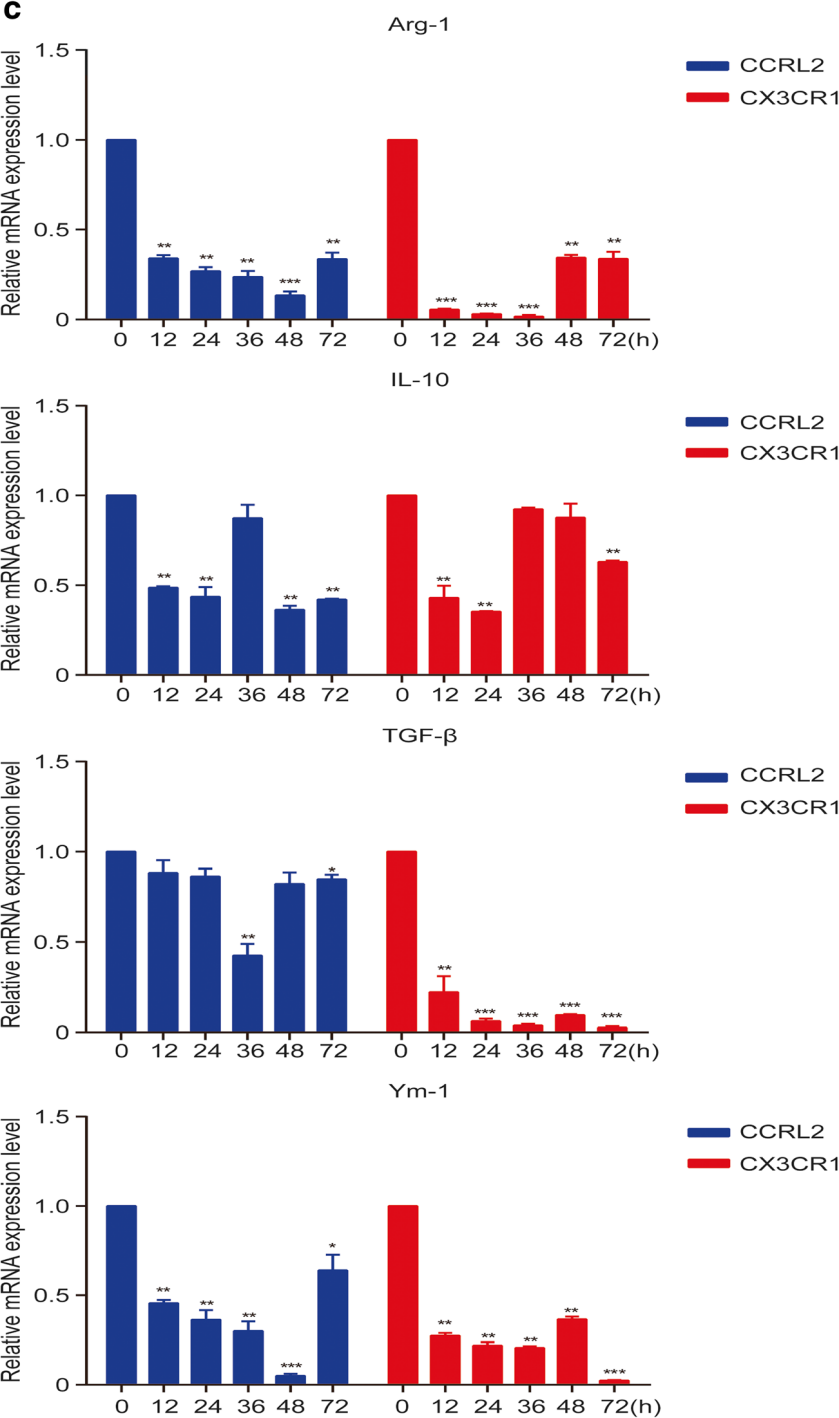

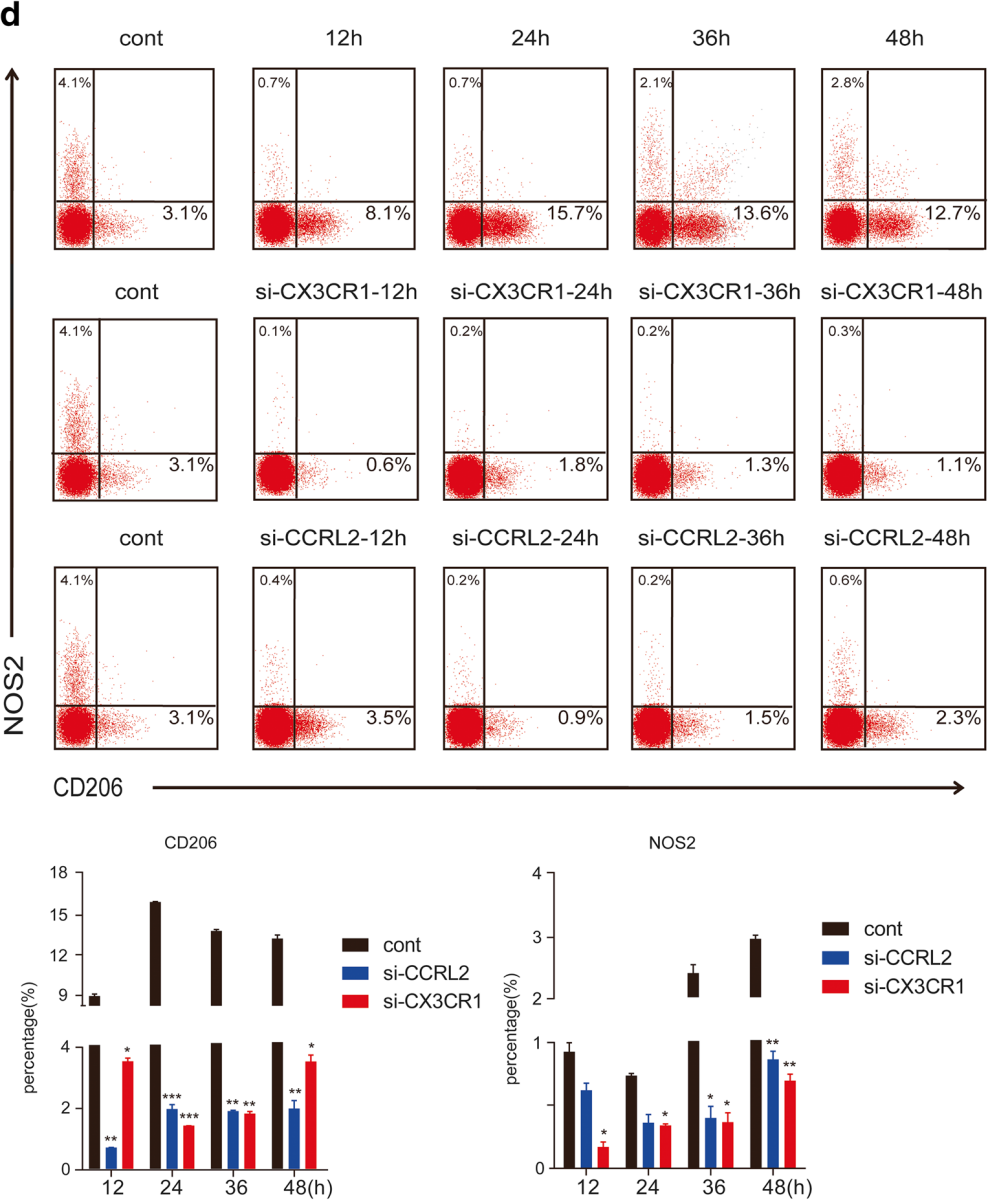

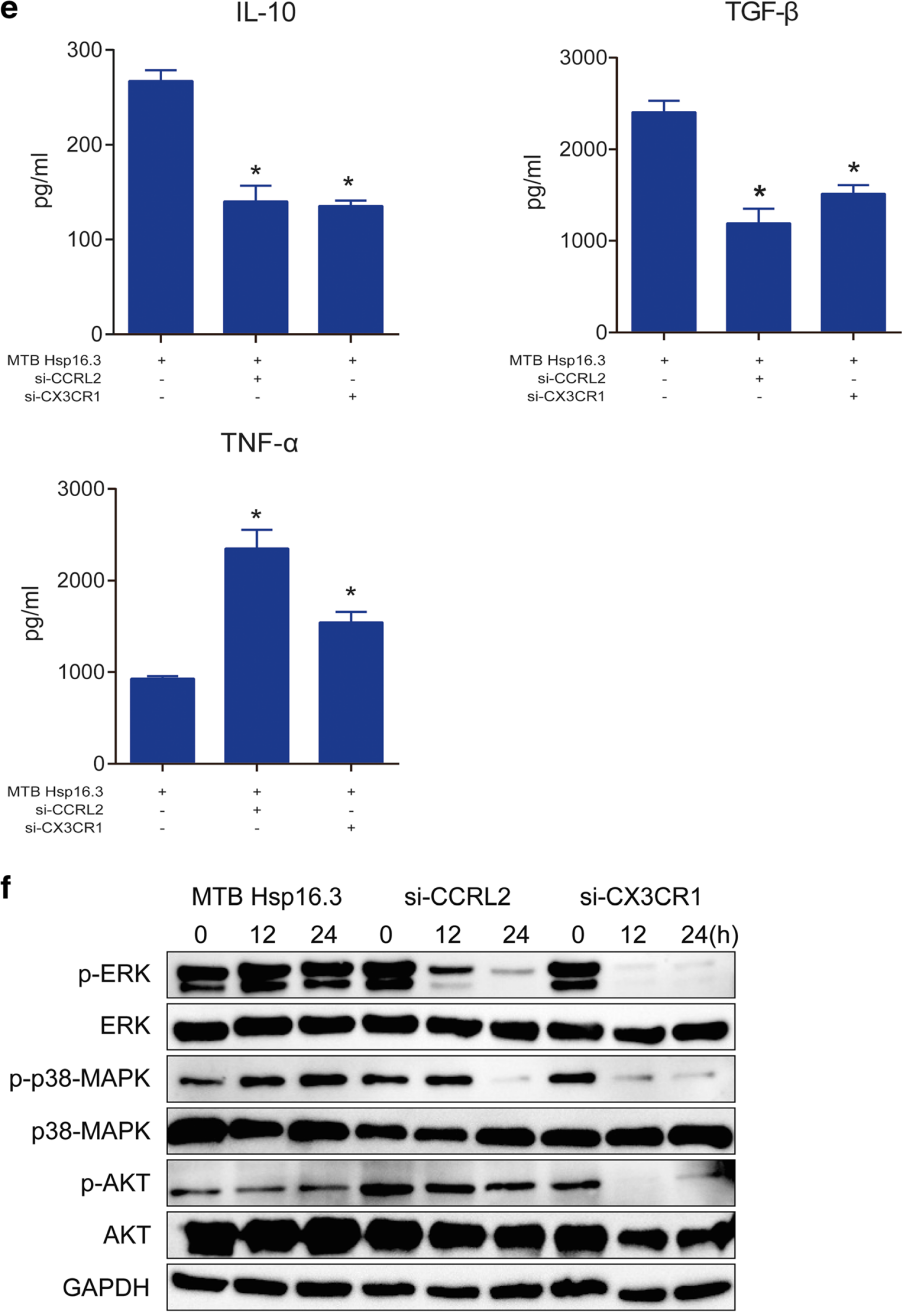
The silencing of CCRL2/CX3CR1 abrogates the MTB Hsp16.3-induced polarization of macrophages to the M2 phenotype. Bone marrow cells were isolated from the tibias and femurs of BALB/c mice (6–8 weeks old) and incubated with 20 ng/ml GM-CSF for 7 days. Then, BMDMs were transfected with siRNA targeting CCRL2 and CX3CR1. **a** mRNA expression levels of CCRL2 and CX3CR1 were measured by real-time PCR after transfection. **b** Western blot analysis of CCRL2 and CX3CR1 in the untreated BMDM group and +Hsp16.3 BMDM group transfected with siRNA targeting mouse CCRL2 and CX3CR1 for 0–48 h. **c** Real-time PCR analysis of mRNA expression levels of Arg-1, IL-10, TGF-β, and YM-1 in untreated BMDMs and Hsp16.3-treated BMDMs transfected with siRNA targeting mouse CCRL2 and CX3CR1 for 0–72 h. **d** FCM analysis of the expression levels of NOS2 and CD206 in the untreated BMDM group and +Hsp16.3 BMDM group transfected with siRNA targeting mouse CCRL2 and CX3CR1 for 0–48 h. **e** ELISA analysis of the secretion levels of IL-10, TGF-β, and TNF-α in the untreated BMDM group and +Hsp16.3 BMDM group transfected with siRNA targeting mouse CCRL2 or CX3CR1 for 48 h. **f** Western blot analysis of p-ERK, ERK, p-AKT, AKT, p-p-38 MAPK, p-38 MAPK in the +Hsp16.3 BMDM group, and +Hsp16.3 BMDM group transfected with siRNA targeting mouse CCRL2 or CX3CR1 at different time points. Data are presented as the mean ± SEM (*n* = 3). *p < 0.05, **p < 0.01, ***p < 0.001 compared with the control group (0 h group).

To further confirm whether CCRL2 and CX3CR1 contribute to the MTB Hsp16.3-induced polarization of macrophages to the M2 phenotype, we constructed sequences to silence CCRL2 and CX3CR1. We found that in the targeting of CCRL2, the silencing efficiency of the second sequence pair was the greatest, whereas the first sequence pair had the greatest CX3CR1 silencing effect (Fig. 3a). The production of CCRL2 and CX3CR1 was significantly suppressed after silencing (Fig. 3b). These results indicate that we silenced CCRL2 and CX3CR1 successfully. Then, we determined the changes in the expression levels of M2 phenotype-related cytokines. The results showed that in the knockdown group, the mRNA expression levels of Arg-1, TGF-β, IL-10, and Ym-1 were inhibited compared to those in the control group (Fig. 3c). NOS2 expression increased, and CD206 expression decreased (Fig. 3d). Compared with the untreated BMDMs, BMDMs transfected with siRNA targeting CCRL2 or CX3CR1 showed significantly decreased production of IL-10 and TGF-β (Fig. 3e) and increased TNF-α production (Fig. 3e) after incubation with MTB Hsp16.3. These results demonstrate that MTB Hsp16.3 may induce BMDM polarization to the M2 phenotype through CCRL2 and CX3CR1.

An increasing body of literature has documented that macrophage polarization is regulated through various signaling pathways, such as the AKT and ERK pathways [17–22]. Thus, to further investigate the downstream signaling in MTB Hsp16.3-induced M2-like macrophages, we analyzed the expression of phosphorylated AKT, phosphorylated ERK, and phosphorylated p38-MAPK in the untreated BMDM group and the +Hsp16.3 BMDM group transfected with siRNA targeting CCRL2 or CX3CR1. The data showed that the levels of p-ERK and p-p38-MAPK were significantly increased in the +Hsp16.3 group (Fig. 3f), whereas the levels were significantly decreased in the +Hsp16.3 BMDM group transfected with siRNA targeting CCRL2; the production of phosphorylated ERK, phosphorylated p38-MAPK, and phosphorylated AKT in the +Hsp16.3 BMDMs transfected with siRNA targeting CX3CR1 was significantly decreased. These results suggest that MTB Hsp16.3 polarizes BMDMs to the M2 phenotype *in vitro* by altering the signal transduction of the AKT/ERK/p38-MAPK pathway.

### CCRL2/CX3CR1 Overexpression Promotes the Macrophage M2 Phenotype

**Fig. 4:**
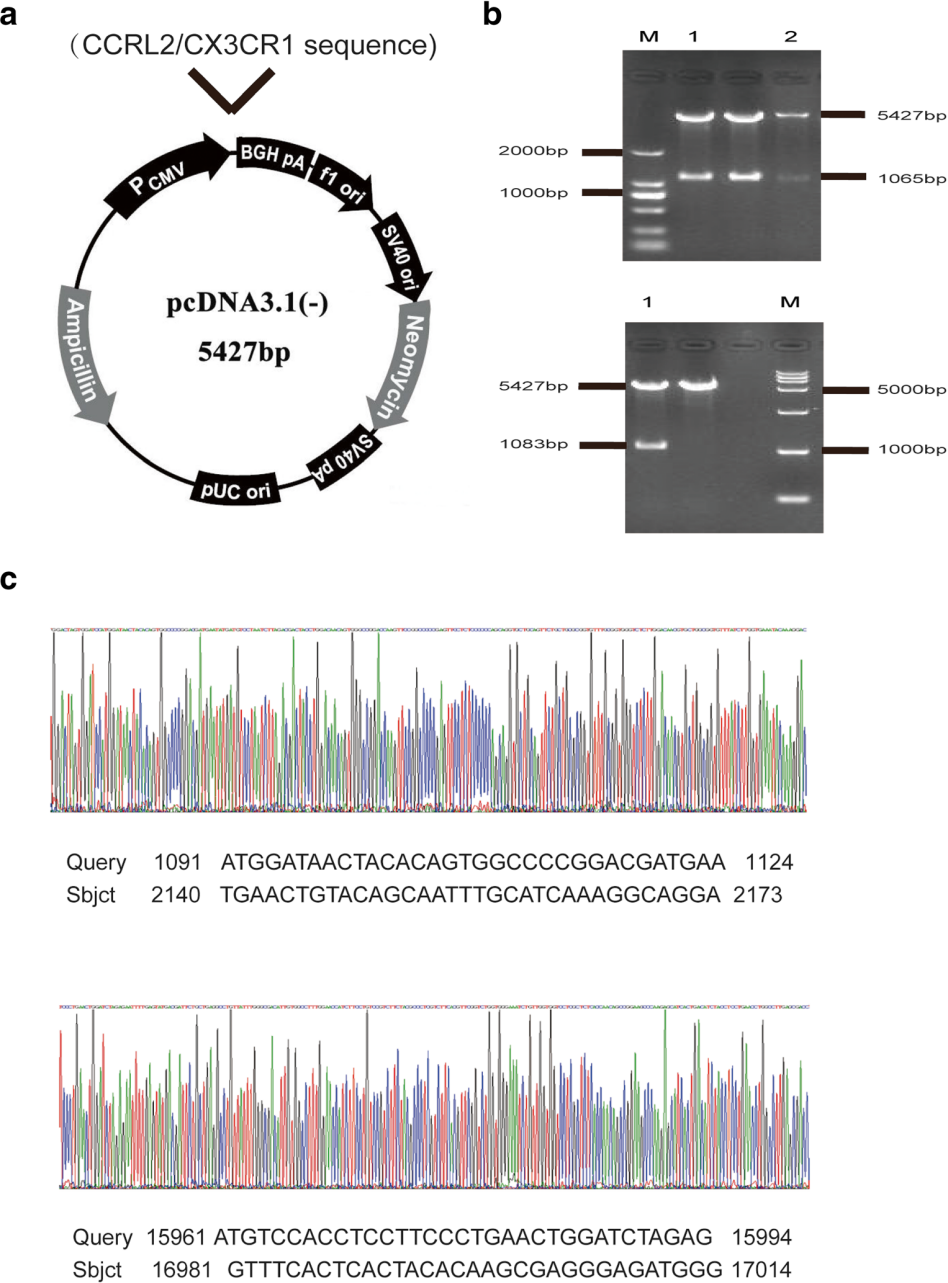

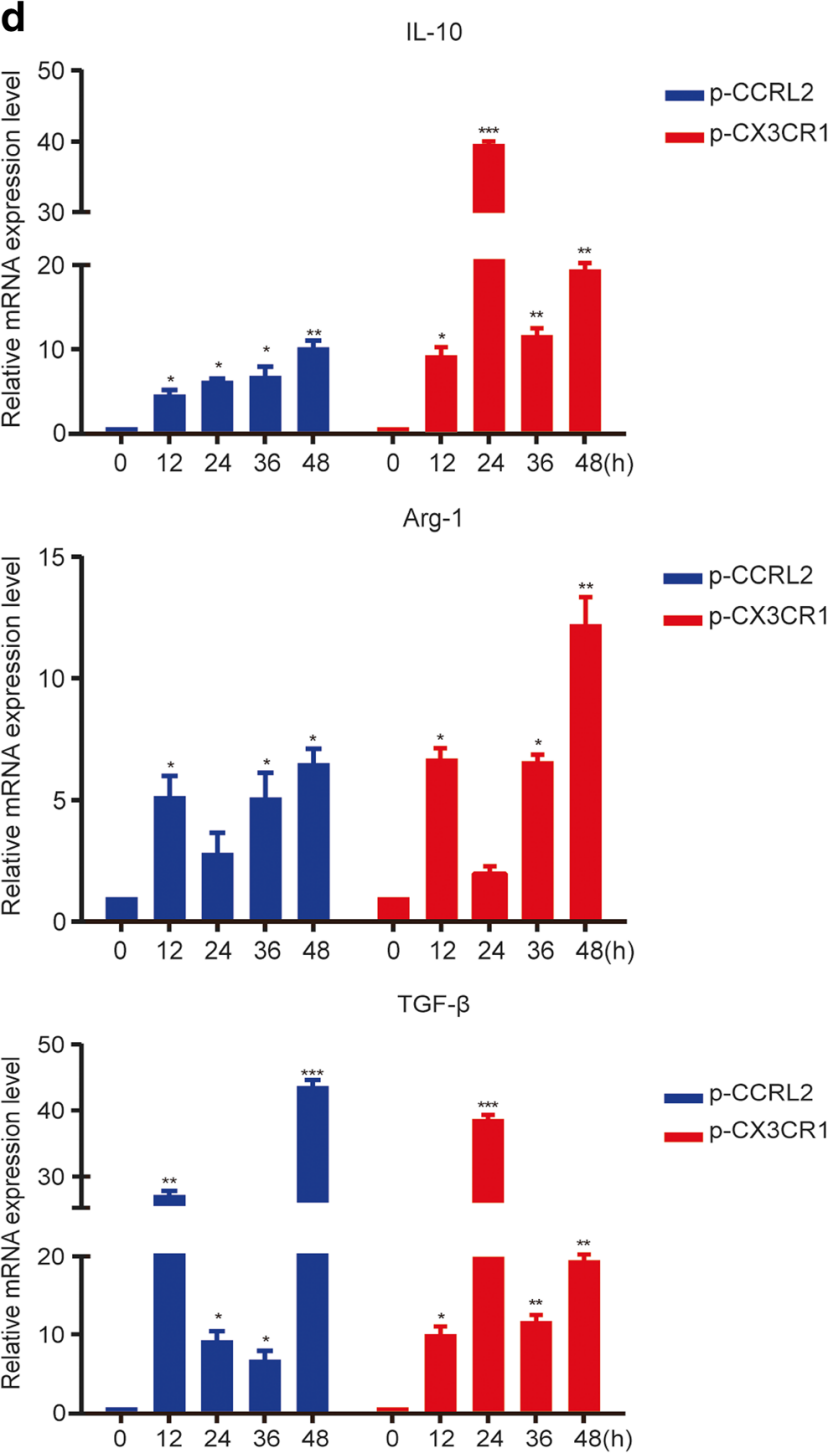

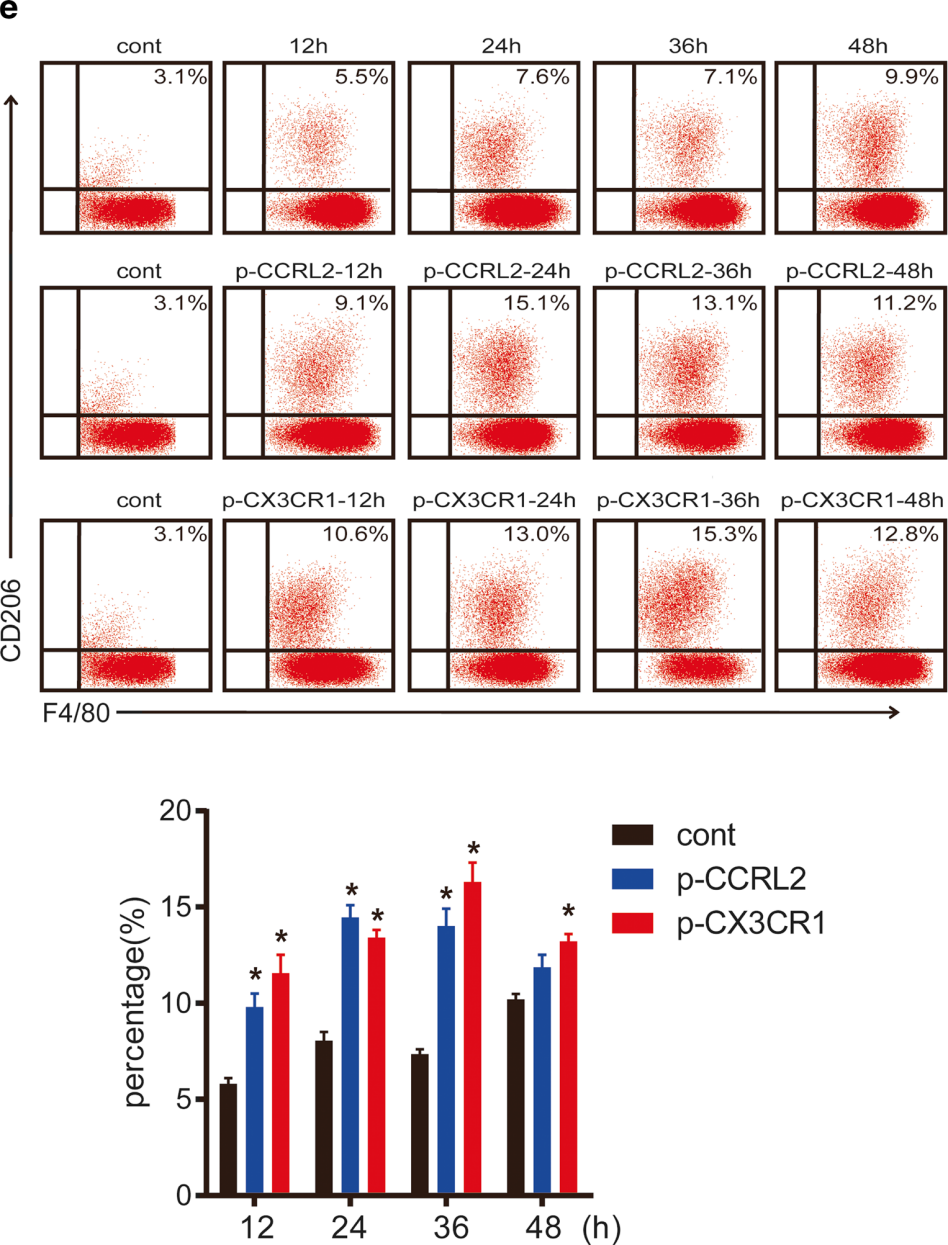

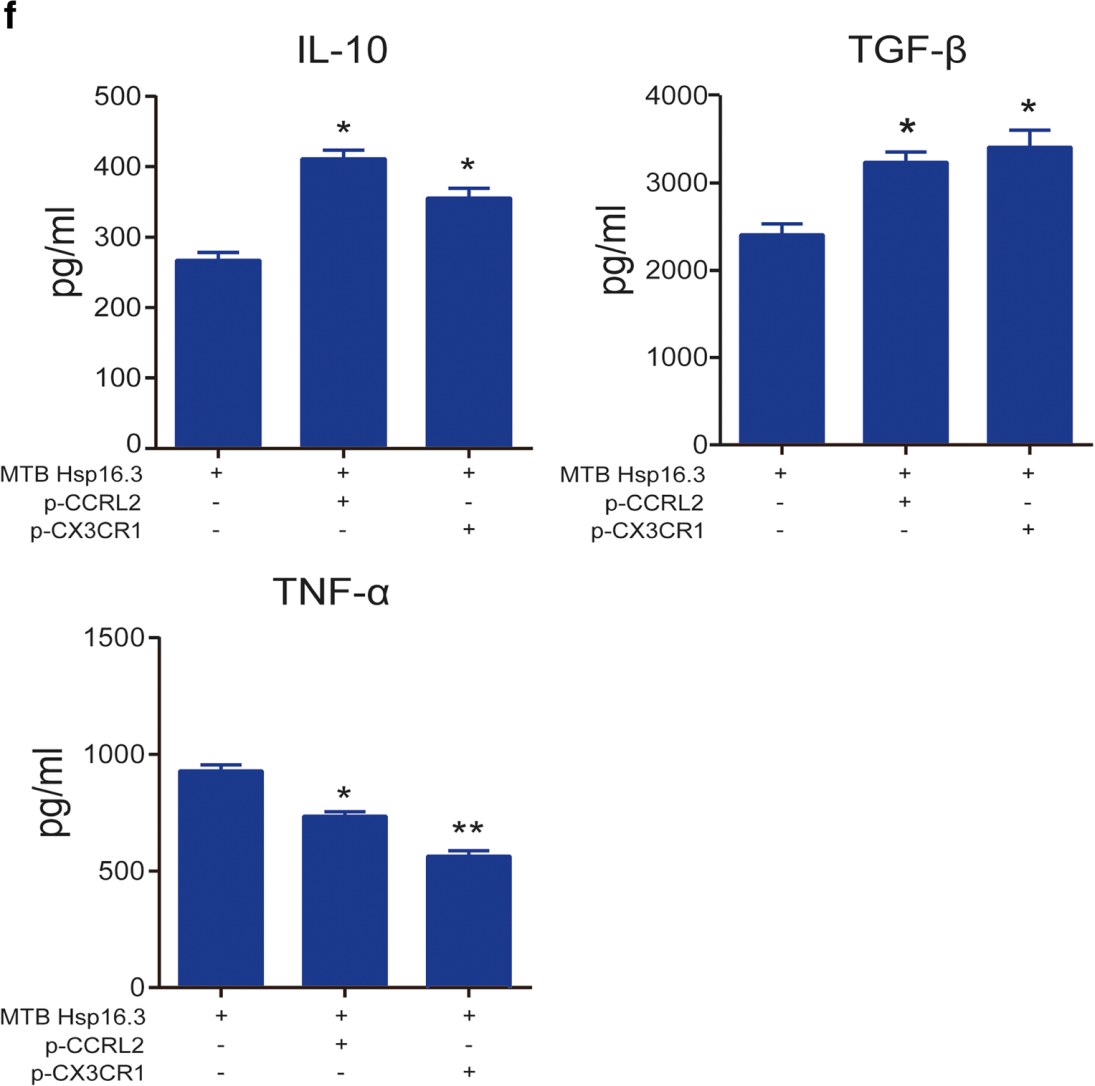
CCRL2/CX3CR1 overexpression promotes the macrophage M2 phenotype. **a** The schematic structure of pcDNA3.1-CCRL2 (termed p-CCRL2) and pcDNA3.1-CX3CR1 (termed p-CX3CR1). **b** Identification of pcDNA3.1-CCRL2 and pcDNA3.1-CX3CR1 by BamHI and HindIII double enzyme digestion. **c** DNA sequence of the recombinant plasmid. BMDMs were transiently transfected with p-CCRL2, p-CX3CR1, or p-cont *in vitro* and then incubated with 100 ng/ml MTB Hsp16.3 for 0–48 h. **d** The mRNA expression levels of IL-10, Arg-1, and TGF-β were determined by real-time PCR. **e** The production of NOS2 or CD206 in F4/80-positive macrophages was measured by FCM. **f** The secretion levels of IL-10, TGF-β, and TNF-α were measured by ELISA at 48 h. The data are presented as the mean ± SEM (*n* = 3). *p < 0.05, **p < 0.01, ***p < 0.001 compared with the control group (0 h group).

To verify the effect of CCRL2 and CX3CR1 on Hsp16.3-induced polarization of macrophages to the M2 phenotype, we constructed pcDNA3.1-CCRL2 (termed p-CCRL2) and pcDNA3.1-CX3CR1 (termed p-CX3CR1) overexpression vectors (Fig. 4a). Two colonies were selected from the positive colonies and cultured in LB medium (+Amp) for 8–12 h, and then the plasmids were extracted. The extracted recombinant plasmids were identified by BamHI and HindIII digestion (Fig. 4b). The results showed that the target bands appeared at 5427 bp, 1065 bp, and 1083 bp after double digestion. The positive plasmid was verified by double restriction enzyme digestion, and the DNA sequence was sent to Hua Da Inc. for complete sequencing (Fig. 4c). The CCRL2 and CX3CR1 sequences in the plasmid were identical to those in the CDS region sequences of CCRL2 and CX3CR1 in the NCBI database. The results above indicate that the p-CCRL2 and p-CX3CR1 overexpression vectors were constructed successfully.

We investigated the M2-related markers to further demonstrate the effect of CCRL2 and CX3CR1 on macrophage polarization. BMDMs were transiently transfected with p-CCRL2, p-CX3CR1, or p-cont *in vitro* for 48 h and then incubated with 100 ng/ml MTB Hsp16.3 for 0–72 h. The results showed that the mRNA expression levels of Arg-1, IL-10, and TGF-β mRNA in the plasmid-transfected groups were significantly upregulated compared with those in the +Hsp16.3 group (Fig. 4d). Compared with the +Hsp16.3 control macrophages, macrophages transiently transfected with p-CCRL2 or p-CX3CR1 incubated with Hsp16.3 showed increased production of CD206 (Fig. 4e), IL-10, and TGF-β (Fig. 4f), and significantly decreased production of TNF-α. Taken together, these results show that MTB Hsp16.3 induces macrophage M2 phenotype polarization through CCRL2 and CX3CR1.

## DISCUSSION

It is well known macrophages activated by danger signals of their own or pathogen sources can be polarized into different subtypes [23]. According to their function, macrophages can be divided into M1 or M2 types. Ml macrophages have enhanced phagocytosis, inflammatory cytokine secretion, and antigen presentation abilities [24]. M2 macrophages can promote wound repair and fibrosis, mediate the escape of tumors and pathogens, and participate in Th2-type immune responses [25]. In long-term latent MTB infections, macrophages lose their ability to present antigens; therefore, T cells do not acquire effector signals to secrete IFN-γ and macrophages are not stimulated. In this microenvironment, macrophages polarized to the M2 phenotype are inhabited by MTB. And many studies have shown that MTB Hsp16.3 expression is increased in the latent infection phase [26], so we surmise that MTB Hsp16.3 might induce macrophage M2 polarization. There were reports about MTB Hsp16.3 inhibiting autophagy and apoptosis [27] but few reports on macrophage polarization. In a previous study, we have expressed and purified MTB Hsp16.3 protein *in vitro* [10]. Based on these findings, in this study, we explored the impact of MTB Hsp16.3 on macrophage polarization.

Our results revealed that MTB Hsp16.3 treatment upregulates the production of M2-related markers in mouse bone marrow-derived macrophages. The secretion of inflammatory cytokines IL-6, TNF-α, and iNOS forms an inflammatory microenvironment that promotes the polarization of macrophages to the M1 phenotype, forming a proinflammatory positive feedback loop. The production of IL-10, TGF-β, and Arg-1 further promotes the polarization of macrophages to the M2 type [28], also forming a positive feedback mechanism for the anti-inflammatory response. Our results revealed that IL-6, TNF-a, iNOS, IL-10, TGF-β, and Arg-1 mRNA expression levels were dynamically changed in macrophages incubated with MTB Hsp16.3 at six time points. We found that the expression changes and morphological characteristics were consistent with those of the M2 phenotype-positive group (macrophages incubated with IL-4). The upregulation of NOS2 expression in macrophages increases the production of NO, which plays a key role in macrophage-mediated pathogen killing. CD206, an M2 marker, can recognize a variety of microorganisms but is often utilized by intracellular pathogens such as *Mycobacterium tuberculosis* for their own survival [11]. Moreover, NOS2 is often regarded as a classical marker of M1 macrophages, while CD206 is an M2 marker [29]. Our data showed that NOS2 expression in the MTB Hsp16.3-treated group was significantly downregulated compared with that in the control group, and the expression of CD206 was upregulated. These results suggest that MTB Hsp16.3 can induce the expression of M2-associated markers in macrophages and create an immunosuppressive microenvironment. Taken together, these results show that MTB Hsp16.3 induces macrophage polarization to the M2 phenotype.

Our data suggest that MTB Hsp16.3 treatment upregulated the production of chemokine receptors CCRL2 and CX3CR1 in BMDMs. Chemokines and their receptors play a vital role in immune surveillance and inflammatory responses as well as in the regulation of angiogenesis, organ formation, tumor growth, and metastasis. CCRL2 and CX3CR1 are chemokine receptors and are adjacent to the CCR gene family [30, 31]. CCRL2, a 7-transmembrane protein, is expressed by most leukocyte subsets, including activated monocyte/macrophages, neutrophils, dendritic cells, lymphocytes, and NK cells [32, 33]. CCRL2 is defined as an “atypical chemokine receptor” (ACKR), which lacks classical GPCR signaling and chemotactic activity and has been shown to limit inflammation through its ability to clear chemokines in areas of inflammation. In addition, CCRL2 is critical for the development of Th2 responses [34], and it has been reported that CCRL2 is associated with macrophage M2 polarization [35]. CX3CR1, the receptor of fractalkine, is also an important chemokine receptor for macrophages. CX3CR1 mediates monocyte patency in the vascular space under steady-state condition, recruits tissue-resident macrophages [29], and regulates tissue macrophage function during various disease processes. Among the innate immune cells, macrophages expressing the chemokine receptor CX3CR1 contribute to maintaining the inflammatory response balance in the gut by producing the anti-inflammatory cytokine IL-10 [36]. In addition, CX3CR1 ablation inhibits the macrophage-mediated repair of acute skeletal muscle damage [37]. Therefore, CX3CR1 may be involved in macrophage M2 polarization. However, there is also evidence that CX3CR1 promotes macrophage M1 polarization [38, 39]. Therefore, the effect of CX3CR1 on macrophage polarization is not clear. In our work, we used a cDNA microarray to analyze the differential expression of genes between the +Hsp16.3 group and the untreated group, and we consulted the relevant literature to identify CCRL2 and CX3CR1, which may be involved in macrophage polarization. We found that MTB Hsp16.3 treatment increased the expression of CCRL2 and CX3CR1 at 3–6 h. Overexpression and silencing techniques further validated that MTB Hsp16.3 induces macrophage M2 polarization *via* CCRL2 and CX3CR1. In addition, we explored downstream signaling molecules affected by CCRL2 and CX3CR1. Because a large number of studies have shown that the AKT/ERK/p38-MAPK signaling pathway plays an important role in the expression of inflammatory factors [40, 41], phagocytosis [42], and resistance to pathogenic bacteria [43, 44], we investigated these signaling molecules. After CCRL2 silencing, p-ERK and p-p38 levels were significantly reduced, and there was no clear change in p-AKT levels. After CX3CR1 silencing, the production of p-ERK, p-p38-MAPK, and p-AKT was significantly reduced. These findings suggest that the MTB Hsp16.3-induced M2 polarization of mouse bone marrow-derived macrophages occurs *via* AKT/ERK/p38-MAPK. In future work, we will provide sufficient proof to indicate how MTB Hsp16.3 interacts with CCRL2/CX3CR1 to induce macrophage M2 polarization, and the downstream signaling will be further verified by blocking the AKT/ERK/p38-MAPK signaling pathway.

In conclusion, we found that MTB Hsp16.3 promotes the polarization of BMDMs to the M2 phenotype *via* CCRL2/CX3CR1 and may be mediated by the AKT/ERK/p38-MAPK signaling pathway. Our results might explore the potential role of Hsp16.3 in LTBI. And we believe an in-depth understanding of the relationship between the polarization of macrophages and MTB interactions will help to improve tuberculosis prevention and control.
